# Smartphone-Based Detection of Potentially Critical Cycling Manoeuvres for Proactive Cycling Safety Assessment

**DOI:** 10.3390/s26165138

**Published:** 2026-08-14

**Authors:** Jörg Ehlers, Alvaro García-Hernández, Arno Wolter

**Affiliations:** 1 Institute of Highway Engineering, RWTH Aachen University, 52074 Aachen, Germany; ehlers@isac.rwth-aachen.de; 2 Initiative for Safer Roads, Matthias-Grünewald-Str. 1–3, 53175 Bonn, Germany; a.wolter@sichere-strassen.org

**Keywords:** proactive traffic safety, bicycle traffic, kinematic data, surrogate safety measures, machine learning

## Abstract

Cycling is increasingly promoted as part of sustainable transport strategies, but safety concerns remain an important barrier. Crash-based safety assessment is limited because crashes are reactive indicators and cycling crashes are often underreported. This study evaluates whether smartphone inertial sensor data can be used to detect selected potentially critical cycling manoeuvres that may support proactive safety screening. A two-stage workflow intended for possible future smartphone implementation was evaluated offline. In Stage 1, kinematic thresholds were applied to identify short candidate sensor windows when a high-dynamic manoeuvre was detected. In Stage 2, a machine learning classifier was applied to these candidate windows to classify the manoeuvre type and reduce false positives. The workflow was evaluated using three datasets collected in Germany from 10 riders, including controlled test-track manoeuvres and urban rides in Aachen and Bonn. The dataset included 148 hard-braking and 132 abrupt-swerving manoeuvres. In addition, 45 bicycle-only crash-like reference sequences were collected to compare low-speed impact-like signal patterns. In leave-one-dataset-out evaluation, the Support Vector Machine achieved mean precision–recall areas under the curve of 0.954 for hard braking and 0.884 for abrupt swerving. The corresponding mean F1-scores were 0.924 and 0.876. The offline evaluation showed that smartphone sensor data can support the detection of selected high-dynamic cycling manoeuvres with good classification performance under controlled and semi-controlled conditions. However, most target manoeuvres were controlled or rider-initiated proxy manoeuvres rather than confirmed traffic conflicts. The results should therefore be interpreted as manoeuvre detection, not as direct crash-risk validation. In future naturalistic studies, the workflow could help identify candidate locations where cyclists frequently brake hard or swerve abruptly. These locations can then be prioritised for site inspections, video-based conflict analysis, or comparison with crash records.

## 1. Introduction

Countries worldwide are increasingly promoting cycling as a fundamental component of sustainable transportation solutions. However, safety concerns remain one of the main reasons preventing people from cycling [1]. These concerns stem from road infrastructure often lacking adequate cycling facilities and protective measures for cyclists [2,3]. Addressing this challenge necessitates modifications to current infrastructure. This process can be both resource-intensive and time-consuming. Therefore, it is crucial to optimise this process by proactively identifying high-risk locations for cyclists and implementing appropriate preventive measures.

Since road safety cannot be measured directly in the same way as physical attributes such as length or temperature, crash data often serve as a proxy for road safety [4]. Metrics such as crash rates or the expected number of crashes are commonly utilised to evaluate crash risk [5]. Although crash data have been instrumental in identifying hazardous locations and informing safety interventions, this approach has several drawbacks. Crash-based analyses are predominantly reactive, as interventions can only be implemented after incidents have occurred. Moreover, underreporting of crashes, particularly those involving cyclists, leads to incomplete crash datasets [6]. For instance, studies have shown that up to 98% of cyclist falls remain unreported [7], which can introduce biases in crash datasets for specific road user groups and geographic areas. Additionally, the relatively low frequency of crashes makes it statistically challenging to confidently identify truly unsafe road sections and to implement preventive measures effectively [8].

To overcome these limitations, alternative methods that do not rely solely on crash data have been developed. Surrogate Safety Measures (SSMs) are metrics based on the premise that there is a relationship between the frequency and severity of traffic events [9]. This approach allows for conclusions about traffic safety to be drawn from less severe incidents that occur more frequently than crashes. Commonly used SSMs include camera-based analyses such as Time to Collision (TTC) and Post-Encroachment Time (PET), which quantify collision risk by evaluating the temporal proximity between road users [10]. However, these camera-based methods are typically limited to fixed locations because they rely on stationary cameras, limiting scalability and thus making network-wide assessments challenging [11].

An alternative approach to network-wide road safety assessment involves the use of vehicle kinematics. By analysing parameters such as braking and steering behaviour, potentially high-risk manoeuvres can be identified across a larger road network [12]. While this methodology has proven effective in motor vehicles, its application to cycling remains limited. One reason is that cycling manoeuvres differ substantially from those of motor vehicles. Bicycles exhibit a greater range of motion and more varied movement profiles, which can complicate direct application of vehicle-based techniques. Moreover, the presence of electrically power-assisted cycles (EPACs) introduces additional heterogeneity; studies indicate that riders of EPACs often travel faster and brake harder than conventional bicycle users, leading to divergent cycling characteristics [13].

Another reason for the limited usage of bicycle-specific kinematic data is that bicycles generally lack the built-in sensors found in modern cars. Researchers have thus employed instrumented bicycles [14,15], helmets equipped with sensors [16], and smartphone sensors [17] to gather cyclist-specific kinematic information. These data have then been used to detect safety-relevant cycling events or high-dynamic manoeuvres through manual annotation [14], heuristic-based methods [17] and machine learning models [18]. However, manual annotation restricts the scalability of such methods across larger networks. Threshold-based detection in naturalistic settings may produce false positives when minor manoeuvres exceed a fixed threshold or false negatives if that threshold is set too high. For machine learning models, a sufficiently large and relevant dataset is also required. Karakaya et al. [18], for example, relied on smartphone-collected data enriched by user-defined event locations and free-text descriptions, which can introduce inaccuracies if users misconstrue an event’s timing or location. More recently, Saleh et al. [19] developed a computationally efficient deep temporal model using the same naturalistic data source, demonstrating continued progress in smartphone-based near-miss detection.

Nevertheless, approaches based on continuously recorded sensor streams may be resource-intensive and raise privacy concerns, particularly if large volumes of raw data are transmitted to a cloud server for processing. Video-based methods can provide valuable contextual information and often yield strong performance [20]. Recent research has further demonstrated the potential of computer-vision frameworks to automate the analysis of cyclist-mounted video and extract environmental, infrastructural and road-user-related risk factors [21]. However, video-based approaches still involve substantial data storage and processing requirements and may also raise privacy concerns when implemented on a large scale. These concerns can discourage long monitoring periods and reduce cyclists’ willingness to participate, as recent research has highlighted in the context of extensive data collection from road users [22].

To address these limitations, this paper presents a two-stage, smartphone-based framework for detecting and classifying potentially critical cycling manoeuvres from inertial sensor data. The framework is intended to support mobile safety screening without relying on stationary camera installations or continuous video recording. It focuses on two high-dynamic manoeuvre types, hard braking and abrupt swerving, which can occur during evasive cycling behaviour and can be observed from ego-bike kinematics. In the proposed future implementation, a lightweight on-device trigger would monitor bicycle kinematics and store short sensor windows when predefined thresholds are exceeded. A cloud-based machine learning classifier would then analyse these candidate windows to classify the manoeuvre type and reduce false positives. The main novelty is the proposed combination of event-triggered data capture and second-stage classification, which is intended to reduce continuous high-frequency data transmission and avoid dependence on user-reported event marking. The proposed workflow is intended to reduce data transmission and support privacy-conscious data collection. Detected manoeuvres should, however, be interpreted as surrogate safety indicators rather than confirmed near-crashes.

## 2. Methodology

### 2.1. Overview of the Methodology

**Figure 1:**
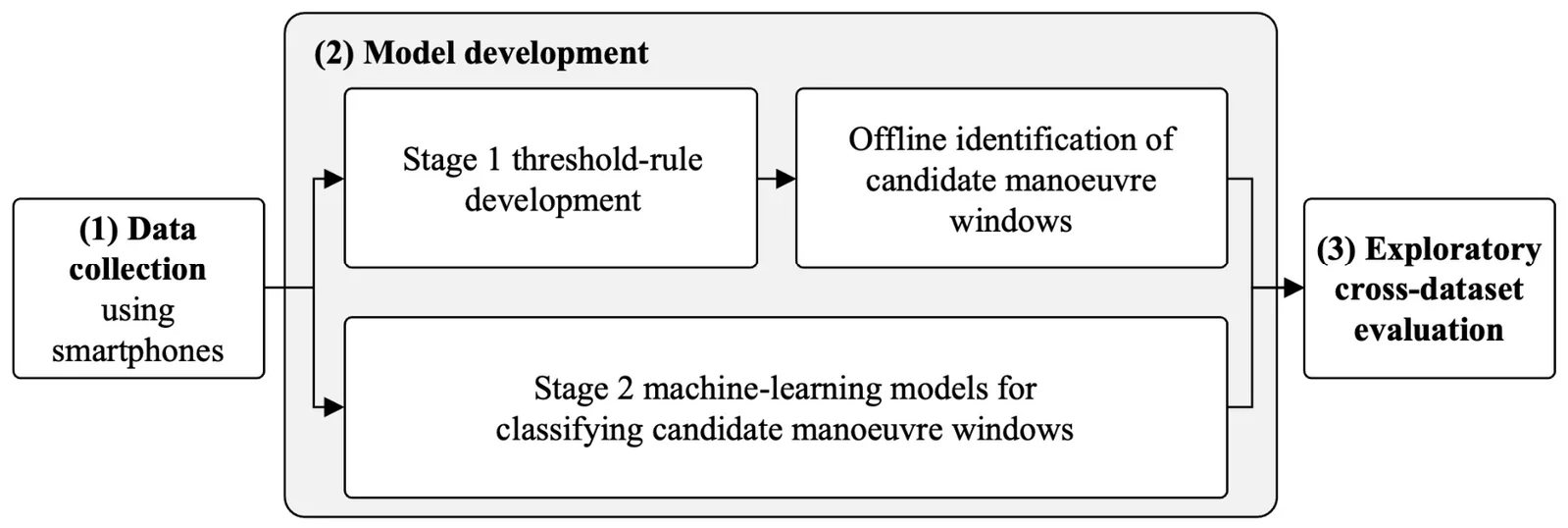
Overview of the methodology for detecting and classifying potentially critical cycling manoeuvres using smartphone data.

Figure 1 provides an overview of the methodology used to develop and evaluate a system for detecting and classifying potentially critical cycling manoeuvres using smartphone data. The approach comprised three main stages: (1) data collection, (2) model development, and (3) offline exploratory cross-dataset evaluation.

A dataset of cycling manoeuvres was collected using smartphones under both controlled and everyday conditions to capture a wide range of riding behaviours. These data included both target manoeuvres and non-critical cycling behaviour and, after labelling and splitting the dataset, formed the basis for developing the detection algorithms.

A threshold-based detection algorithm was derived from decision trees for possible future smartphone implementation. Separate Stage 1 models were trained for hard braking and abrupt swerving. During offline evaluation, these algorithms were applied to identify candidate high-dynamic manoeuvres. Separate Stage 2 binary classifiers were developed for hard braking and abrupt swerving, with multiple model families compared for each target. In the proposed future workflow, Stage 2 would be applied only to candidate windows identified by Stage 1. Overall performance was assessed using an exploratory leave-one-dataset-out cross-dataset evaluation.

### 2.2. Defining Potentially Critical Cycling Manoeuvres

In this study, the target class was defined as potentially critical cycling manoeuvres that can be observed from the bicycle’s kinematic response. These manoeuvres correspond to abrupt changes in speed or trajectory that may occur during evasive behaviour. The definition is inspired by traffic conflict theory, where an evasive reaction by at least one road user is a central element [23]. However, the present study uses ego-bike sensor signals only and does not include contextual information about the surrounding traffic. Therefore, the detected manoeuvres are treated as surrogate safety indicators, not as confirmed near-crashes.

Because genuine near-crash situations are rare and cannot be deliberately provoked for ethical reasons, additional critical-like proxy manoeuvres of hard braking and abrupt swerving were recorded in traffic-free locations to increase the number of target examples (see Section 2.4). These proxy manoeuvres were designed to reproduce the ego-bike kinematic patterns associated with evasive braking and swerving, but they should not be interpreted as confirmed traffic conflicts. For the signal-based classification task, genuine traffic-related manoeuvres and critical-like proxy manoeuvres were assigned to the same target class because the classifier used only ego-bike kinematic signals and did not include contextual information about other road users.

Other safety-relevant situations, such as close passes in which a cyclist is narrowly missed by another road user without a clear evasive response, cannot be reliably captured from ego-bike kinematics alone. These situations were therefore excluded from the present taxonomy. Because this paper focuses on manoeuvres that can be identified from observable kinematic patterns, some hazardous interactions may remain undetected. Future work could address these cases by integrating additional sensors that capture the surrounding traffic environment during cycling.

Crashes were not treated as a target class. Rider-involved crashes are rare and cannot be deliberately collected for ethical reasons. Therefore, low-speed bicycle-only crash-like reference tests were recorded only to compare impact-like signal magnitudes with the target manoeuvres. These reference tests used an unoccupied bicycle and were not used to train a crash-detection model. Crash detection and crash-severity estimation are beyond the scope of this study.

To ensure a representative non-critical class, routine cycling behaviour was also logged. During model training and evaluation, however, all such routine manoeuvres were pooled into a single “non-critical” class; the finer taxonomy served only for internal data-quality checks.

### 2.3. Devices to Collect Kinematic Data During Cycle Rides

#### 2.3.1. Sensor Types and Selection of Smartphone Models

This work used smartphones to collect kinematic data for detecting and classifying cycling events. Five integrated sensors were selected to comprehensively capture sudden changes in movement, the trajectories and smartphone orientation:

- Accelerometer: measures tri-axial acceleration to capture sudden movements.
- Gravity sensor: provides the gravity vector for determining device orientation.
- Linear acceleration sensor: removes gravity from the accelerometer data.
- Gyroscope: measures tri-axial angular velocity to capture rotational movements.
- Global Navigation Satellite System (GNSS): determines the position to link events with specific locations, enabling analysis in the context of infrastructural factors.

The accelerometers and gyroscopes integrated into contemporary smartphones are commonly based on microelectromechanical systems (MEMSs), whereas the gravity and linear-acceleration signals may be derived through sensor fusion, depending on the device [24]. In future large-scale applications, participants would use their own smartphones. These hardware differences may affect sampling rates, noise levels, and thus the overall accuracy of sensor measurements. To account for this diversity when training and testing the classification models, four representative devices were selected based on a database of smartphone usage frequencies and technical specifications [25] to represent a range of market segments and sensor implementations: two devices ran Apple’s iOS operating system (iPhone SE (2020), iPhone 12 (Apple Inc., Cupertino, CA, USA)), whereas two ran the Android operating system (Samsung Galaxy A52 (Suwon-si, Republic of Korea), Xiaomi 13 (Beijing, China)). Additionally, the data processing approach in this work imposed a consistent sampling rate and applied smoothing filters (see Section 2.5) to mitigate potential sensor-related discrepancies across different hardware models.

#### 2.3.2. Placement and Orientation of Smartphones

**Figure 2:**
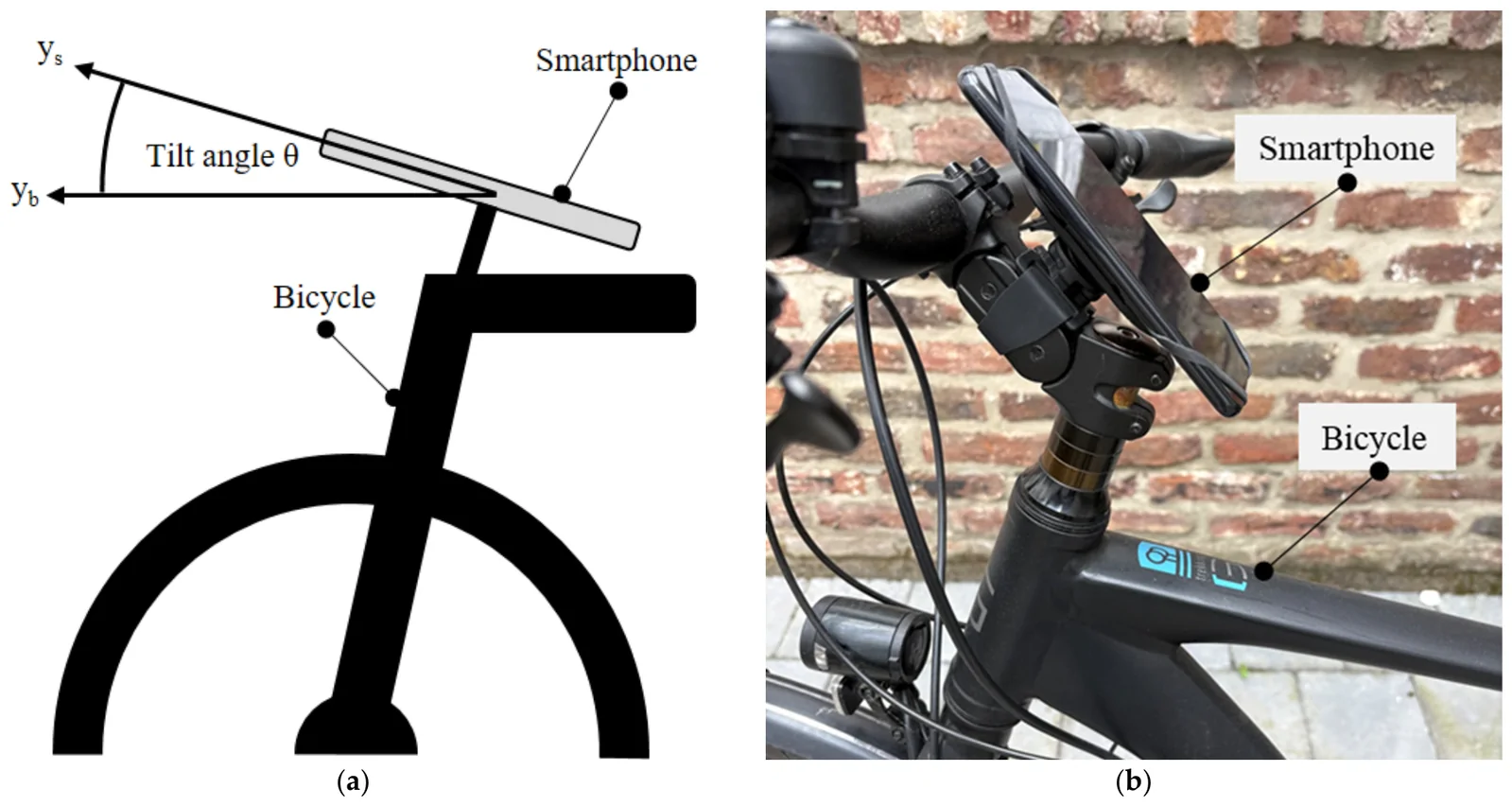
(**a**) The smartphone is placed on the bicycle’s handlebar and is oriented along the bicycle’s longitudinal axis (y_b_), with its own y-axis (y_s_) forming a tilt angle (θ) ranging from 0° to 70° relative to y_b_. (**b**) Exemplary smartphone placement for measuring kinematic data during cycling.

During the cycling rides, the smartphone was mounted on the handlebar and aligned with the direction of travel, with pitch angles ranging from 0° to 70° around its transverse axis (see Figure 2). The coordinate system follows the standard convention for smartphones [26]: in an upright orientation with the screen facing the rider, the x-axis points to the right side of the device, the y-axis points upward, and the z-axis projects outward from the display toward the rider.

All smartphones were placed upright to standardise mounting across users and ensure consistent sensor data. The variation in pitch angles was introduced deliberately to cover differences in mounting angles within the intended standardised upright handlebar configuration. Without adjusting for these differences, the sensor readings would not be directly comparable across varying device placements. A pitch rotation around the transverse x-axis does not alter the lateral x-component, but it redistributes the longitudinal and vertical components between the smartphone’s y- and z-axes. Consequently, the same bicycle motion can produce different signal amplitudes on these two device axes depending on the mounting angle. To account for this effect, a coordinate transformation was applied to rotate the acceleration and angular velocity measurements from the smartphone’s frame of reference into the bicycle frame of reference before feature extraction and model development.

The tilt angle *θ* between the smartphone and the horizontal plane was determined using Equation (1):

(1) $$\theta =atan2(G_{y},G_{z}),$$

where *atan*2 is the four-quadrant inverse tangent and *G_y_* and *G_z_* are the components of the gravitational force along the y_s_- and z_s_-axes of the device, respectively.

To express sensor data in the bicycle’s frame of reference, the transformation in Equation (2) was applied:

(2) $$\left(\begin{array}{c} x_{b}\\ y_{b}\\ z_{b} \end{array}\right)=\left(\begin{array}{ccc} 1 & 0 & 0\\ 0 & cos(\theta ) & -sin(\theta )\\ 0 & sin(\theta ) & cos(\theta ) \end{array}\right)\left(\begin{array}{c} x_{s}\\ y_{s}\\ z_{s} \end{array}\right),$$

where *x_b_*, *y_b_*, and *z_b_* are coordinates in the bicycle’s frame of reference; *x_s_*, *y_s_*, and *z_s_* are coordinates in the smartphone’s frame of reference; and *θ* is the tilt angle around the transverse axis. This alignment was applied to both acceleration and angular velocity measurements across all recordings. Because pitch was treated as a mounting-related variation accounted for before feature extraction, model performance was not evaluated separately by pitch angle.

The transformation corrected only the pitch misalignment. Roll (rotation around the longitudinal axis) and yaw (rotation around the vertical axis) were not estimated dynamically. Instead, these offsets were controlled through the experimental mounting procedure: all smartphones were mounted upright in a stable handlebar holder and aligned using a photo-based mounting guide. Under these conditions, roll and yaw misalignment were assumed to be small for the purposes of the kinematic analysis. Nevertheless, residual roll or yaw misalignment could mix the axis components within the acceleration and angular-velocity measurements, potentially affecting the lateral acceleration and yaw rate features used for classification. The present results are therefore restricted to this defined mounting configuration and do not demonstrate robustness to arbitrary smartphone orientations.

For less controlled crowdsourcing or unrestricted bring-your-own-device applications, a full three-axis orientation-alignment procedure would be required to transform measurements from the smartphone coordinate system into the bicycle coordinate system. This could be implemented using a rotation-matrix-based or quaternion-based transformation, together with a short calibration manoeuvre to estimate the smartphone’s orientation relative to the bicycle and compensate for roll and yaw offsets. Model performance should subsequently be assessed under systematic mounting offsets.

#### 2.3.3. Sampling Rate and Data Collection Software

To ensure that all sudden movements were adequately recorded during the initial data collection for model development, the kinematic sensor signals were sampled at 100 Hz. Other studies have recorded similar data at frequencies ranging from 1 Hz [27] to 100 Hz [14], indicating that an optimal sampling rate has not yet been definitively established. Consequently, a high sampling rate of 100 Hz was chosen to effectively capture rapid changes in motion.

Due to hardware limitations, the GNSS was sampled at 1 Hz, which was the maximum sampling frequency supported by the smartphones. Because the GNSS was primarily used to provide approximate location references for future spatial analyses of road infrastructure, its lower frequency did not affect the performance of the classification models. Future iterations of this approach could adopt more precise positioning methods if higher-resolution trajectory information becomes necessary.

For data collection, the smartphone application Phyphox was utilised. Phyphox is an open-source application that grants access to raw sensor data [28]. This allowed the required measurements to be recorded consistently on all smartphone devices during the development phase.

### 2.4. Data Collection for Potentially Critical Manoeuvre Detection

Three complementary datasets were collected using the smartphone instrumentation described in Section 2.3. The sets differed in environment, geographic area and rider population. Table 1 summarises their key characteristics.

Before feature extraction, recordings were screened for sensor-stream interruptions. Four Bonn hard-braking recordings contained sensor-data loss and were excluded. Accordingly, 37 Bonn hard-braking manoeuvres and 148 hard-braking manoeuvres overall were retained. The excluded recordings were not used for feature extraction, model training, hyperparameter tuning, cross-validation or held-out evaluation. All counts reported below refer to the retained quality-controlled data.

A track-controlled set with clean reference manoeuvres is described in Section 2.4.1. Two urban datasets from different cities captured quasi-naturalistic variability across riders and traffic environments and are detailed in Section 2.4.2. The three datasets comprised mutually exclusive rider groups. No participant contributed data to more than one dataset. All datasets were recorded with the same smartphone instrumentation and labelled according to the manoeuvre taxonomy in Table 2, which enabled their joint use for model development and cross-dataset assessment.

#### 2.4.1. Data Collection Under Controlled Conditions

Controlled manoeuvres on a test track were conducted to capture clean kinematic patterns of both target manoeuvres and non-critical cycling behaviour while prioritising safety. All manoeuvres listed in Table 2 were investigated, including the two target manoeuvre types, hard braking and abrupt swerving. In addition, bicycle-only crash-like reference tests were conducted.

Hard-braking trials were conducted at speeds ranging from 10 to 25 km/h and required rapid deceleration to either a complete stop or a speed reduction of 10 and 15 km/h in order to reproduce strong braking patterns that can occur during evasive behaviour. Braking intensity was varied from strong decelerations to maximum braking to cover a range of high-dynamic braking patterns. In addition, non-critical braking manoeuvres were executed within the same speed range to replicate everyday deceleration, such as slowing for traffic or approaching a stop sign.

Abrupt swerves were carried out at speeds ranging from 10 to 25 km/h, with evasive actions performed to both the left and the right. Steering intensity and speed were varied to cover a spectrum from mild to extensive lateral deviations. Standard left and right turns and lane changes were performed to represent typical steering manoeuvres during regular cycling. These turns were also executed at speeds of 10 km/h to 25 km/h and served as baseline data for comparison with abrupt-swerving manoeuvres.

All manoeuvres were conducted on obstacle-free asphalt surfaces, including flat terrain and a 3% slope. Although the surface was free of major defects, it still featured minor irregularities typical of public road surfaces, ensuring realistic vibration levels. Trials were conducted in daylight and under dry weather conditions to maximise visibility and traction. Four experienced cyclists from the project team carried out all manoeuvres. Each rider completed 8 to 12 hard braking and 8 to 16 abrupt swerving trials, yielding 58 hard-braking and 58 abrupt-swerving samples. Minor variation in counts occurred because of rider availability. The chosen number of repetitions balanced the need for sufficient data against practical constraints, including physical fatigue resulting from repeated braking and acceleration.

To obtain reference signals for impact-like kinematic patterns, a series of bicycle-only crash-like reference tests was performed. In these tests, an unoccupied bicycle was propelled on a flat asphalt surface at approximately 5 km/h and directed against a wall and a kerb at both 90° and 45° angles from the left and right. The bicycle was also allowed to fall over on a flat surface and on a 2% slope. Each of the 9 scenarios was repeated five times, resulting in 45 reference sequences. No rider was involved for safety reasons. These recordings were not intended to represent rider-involved crash dynamics and were not used as a crash target class. They served only as reference signals for comparing low-speed impact-like patterns with the hard-braking and abrupt-swerving manoeuvres. Future studies could use a dummy or a rigid body to better approximate the mass and inertial characteristics of the rider–bicycle system, including the height of the centre of gravity and the distribution of load between the front and rear wheels.

#### 2.4.2. Data Collection Under Uncontrolled Conditions

Track experiments cannot reproduce the diversity of surface types, infrastructure layouts and cycling styles encountered in everyday cycling. To capture such variability and to obtain additional high-dynamic manoeuvre signals in urban riding contexts, kinematic data were recorded during regular bicycle rides in two German cities, Aachen and Bonn.

In each city, three project-team riders collected the data. Each group used one electrically power-assisted bicycle and two conventional bicycles, all equipped with the handlebar-mounted smartphone instrumentation described in Section 2.3 to ensure consistency with the controlled test-track recordings. In total, 45 trips were recorded: 30 in Aachen and 15 in Bonn. During these trips, the riders followed normal urban routes and cycled in their usual manner. At self-selected traffic-free locations, such as empty side streets or cycle paths, they additionally performed deliberate hard-braking and abrupt-swerving manoeuvres. The urban datasets therefore contained two types of data: routine riding segments recorded in authentic urban environments and rider-initiated critical-like proxy manoeuvres recorded under safe traffic-free conditions. This design allowed high-dynamic ego-bike kinematic patterns to be captured without exposing riders or other road users to additional risk. These proxy manoeuvres should be interpreted as signal-based target examples, not as confirmed traffic conflicts.

Each ride was filmed with a secondary smartphone camera that was mounted on the handlebar and tilted downward so that only the front tyre was in focus. The footage was used exclusively to label the kinematic sensor data according to the manoeuvre taxonomy in Table 2. After the labelling process had been completed, the raw video files were deleted. Only the derived event-label files and the corresponding kinematic signal files remained in the dataset.

This procedure yielded 53 hard-braking and 48 abrupt-swerving target manoeuvres in Aachen and 37 hard-braking and 26 abrupt-swerving target manoeuvres in Bonn; no crashes occurred. Only three hard-braking manoeuvres were genuine traffic-related events. All other target manoeuvres in the urban datasets were deliberately performed by the riders on traffic-free stretches, as described above. Segments without flagged target manoeuvres were used as non-target baseline data.

The urban datasets involved six riders and were not intended to represent the demographic and behavioural diversity of the general cycling population. This study was designed to assess the technical feasibility of detecting predefined kinematic manoeuvres under semi-controlled urban conditions. The findings therefore do not establish population-level generalisability across rider age, cycling experience, or bicycle type. Future studies should include a larger and more diverse participant sample and systematically assess performance for different rider and bicycle groups.

Because participants followed their usual routes, the recordings included naturally occurring variation in pavement types and riding environments. However, pavement and weather conditions were not systematically documented, balanced, or evaluated as separate experimental factors. The present study therefore does not establish surface- or weather-specific model performance. Future validation should systematically compare defined pavement types and weather conditions, including wet surfaces.

### 2.5. Sensor Data Preprocessing

**Figure 3:**
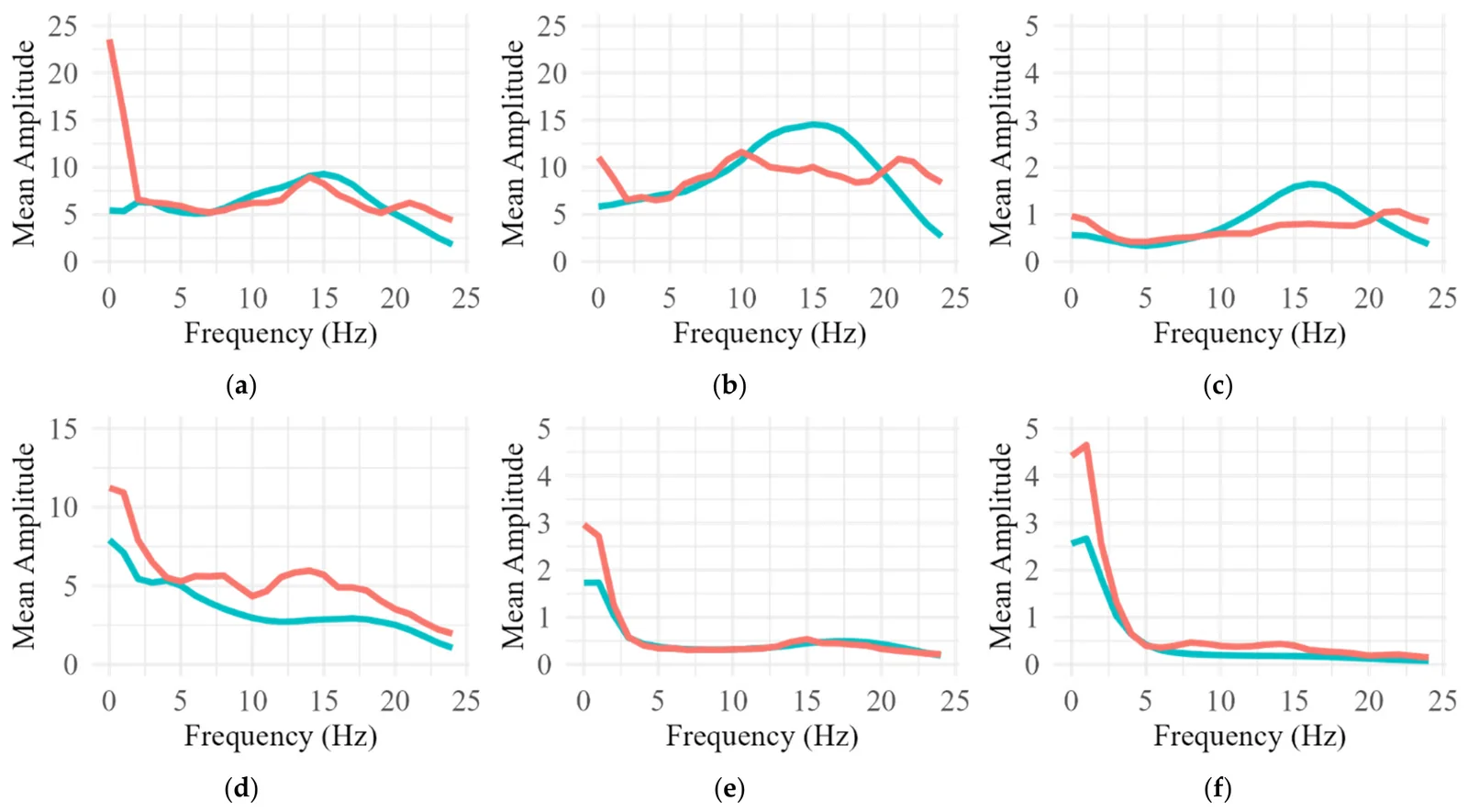
Mean amplitude spectra of selected signal channels for target and non-target manoeuvres. Red lines represent target manoeuvres and blue lines represent non-target cycling behaviour. Panels (**a**–**c**) show hard-braking target manoeuvres, whereas panels (**d**–**f**) show abrupt-swerving target manoeuvres, each compared with non-target cycling behaviour. The signal channels are: (**a**) longitudinal acceleration, (**b**) vertical acceleration, (**c**) angular velocity around the x-axis, (**d**) lateral acceleration, (**e**) angular velocity around the y-axis, and (**f**) angular velocity around the z-axis.

Raw sensor data contain noise originating from the road surface, the smartphone mount, and device-intrinsic electronic noise. To select an appropriate smoothing filter, the frequency components relevant for distinguishing target from non-target manoeuvres were analysed using a short-time Fourier transform with a 1 s window and 50% overlap. Figure 3 shows the mean amplitude spectra for target and non-target manoeuvres across the sensor channels with the largest class-separating differences. For clarity, the frequency range is shown from 0 to 25 Hz, where the main differences between target and non-target manoeuvres were observed.

The spectra show that the highest mean amplitudes and the most pronounced differences between target and non-target manoeuvres occurred predominantly below 2 Hz. A higher-frequency component around 15–17 Hz was also observed in three channels. This component was well above the dominant frequency range of the voluntary manoeuvre dynamics examined here and was therefore interpreted as vibration-related signal content. Its physical origin was not isolated in this study and may reflect excitation within the coupled bicycle-holder-smartphone system, road-surface input, or vibration associated with bicycle structure and suspension. Future controlled experiments using different smartphone holders, mounting stiffnesses, bicycle types, suspension configurations, and road surfaces could help identify the source of this component.

A moving-average filter with a 0.5 s window was therefore applied to each accelerometer and gyroscope axis. This reduced rapid fluctuations in the signals while preserving the overall manoeuvre patterns associated with braking and swerving. Other filtering methods, such as Butterworth or frequency-domain filters, may also be suitable. Future work could examine whether these alternatives provide additional benefits for classification performance.

**Figure 4:**
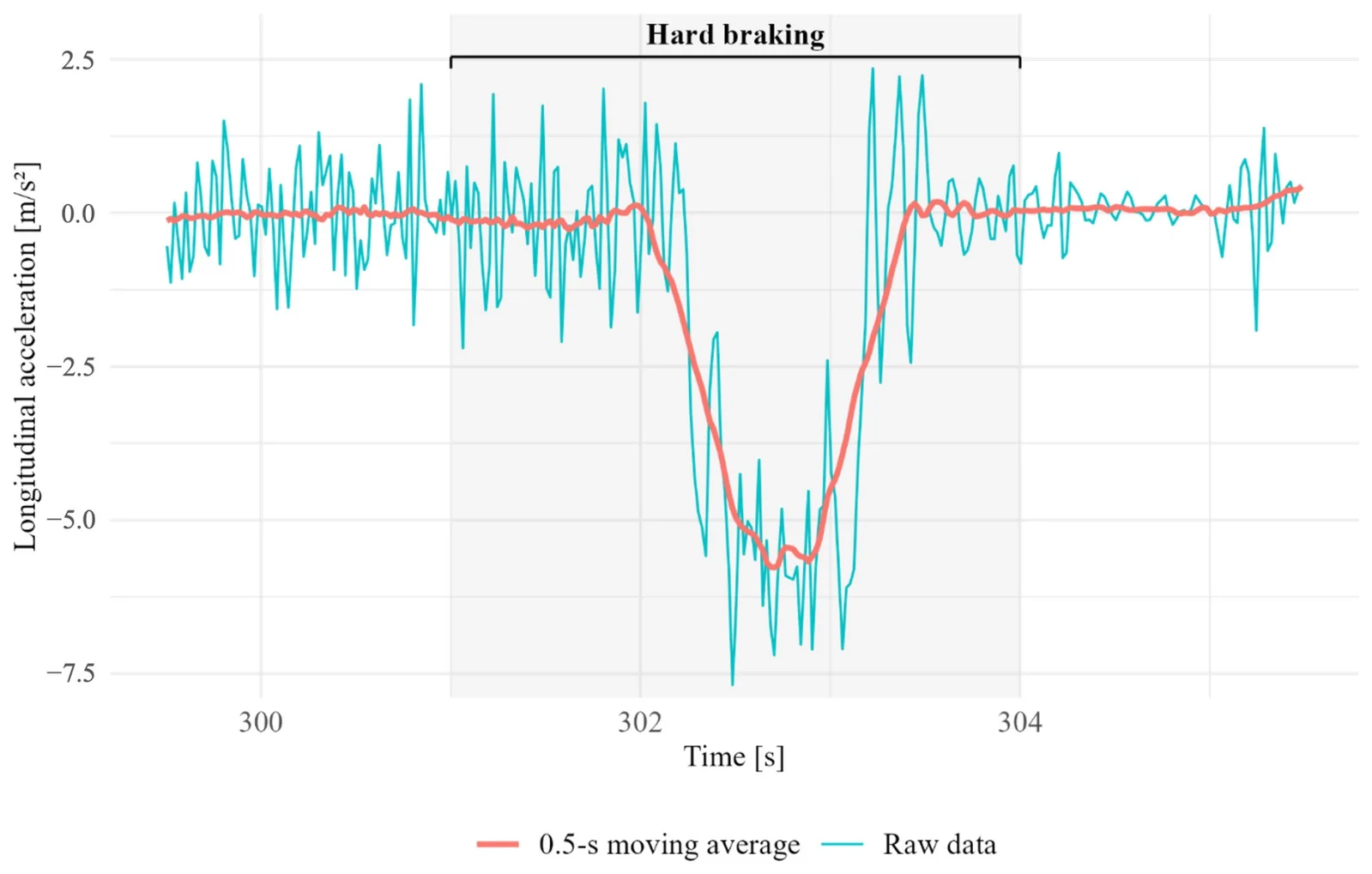
Comparison of raw and filtered longitudinal acceleration data. The 6 s segment contains a 3 s hard-braking manoeuvre, indicated by the grey background.

Figure 4 illustrates the effect of the filter on the longitudinal acceleration signal for a representative 6 s segment that includes a 3 s hard-braking manoeuvre, shown by the grey background, and normal riding before and after the manoeuvre. The filtered trace retains the shape and magnitude of the hard-braking manoeuvre, whereas high-frequency fluctuations in the raw signal are attenuated by the filter.

### 2.6. Detection and Classification of High-Dynamic Cycling Manoeuvres

#### 2.6.1. Overview of the Two-Stage Workflow

The proposed detection workflow was developed to identify candidate high-dynamic cycling manoeuvres while minimising the amount of stored sensor data. It was designed for future naturalistic cycling studies in which participants could use the smartphone application during their routine rides.

**Figure 5:**
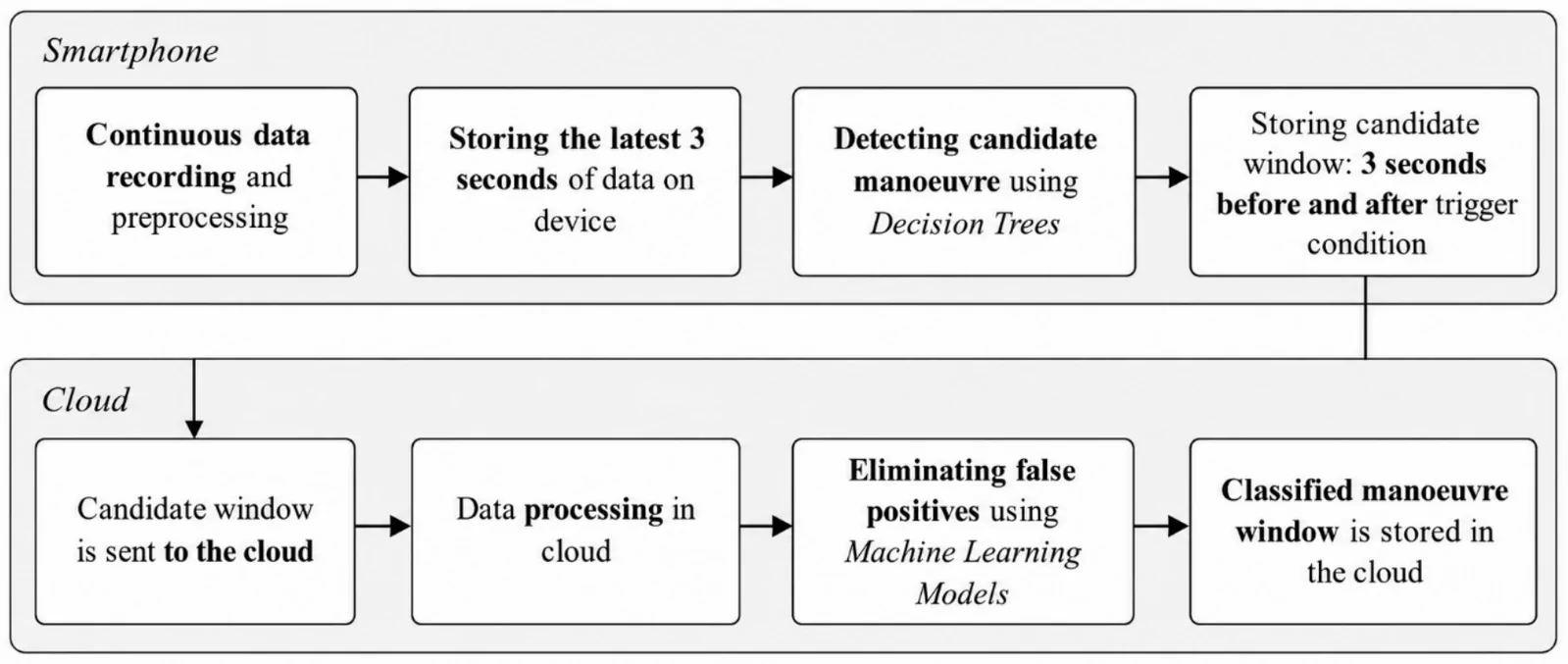
Proposed future two-stage smartphone–cloud workflow for recording candidate manoeuvre windows and classifying potentially critical cycling manoeuvres in naturalistic cycling studies.

The proposed workflow follows a two-stage approach, as illustrated in Figure 5. In a possible future implementation, Stage 1 would use a threshold-based algorithm to monitor smartphone sensor signals. Whenever predefined thresholds were exceeded, it would retain the preceding three seconds and the subsequent three seconds of sensor data, producing a six-second candidate window. This setup is conceptually similar to that used by Liu et al. [29], who recorded three seconds before the first threshold crossing and three seconds after the final threshold crossing. However, preliminary analyses of the collected data indicated that each target manoeuvre was fully contained within a six-second window. A variable-length window would therefore increase the amount of stored data and the risk of including unrelated signal segments, while providing no clear benefit for detecting these manoeuvres. Therefore, a fixed six-second window was selected. In the proposed future workflow, the stored candidate windows would then be transmitted to a cloud server, where the Stage 2 models would classify the manoeuvre type and reduce the number of false-positive candidates generated by Stage 1.

In a future implementation, processing data on the device and transmitting only short candidate windows would reduce data transfer and computational requirements on the mobile device. The present study evaluated this workflow offline; real-time smartphone operation was not tested.

#### 2.6.2. Dataset Configuration for Model Training and Exploratory Cross-Dataset Evaluation

Within each leave-one-dataset-out configuration, one complete dataset was reserved for testing (see Table 3). Because the three datasets contained mutually exclusive rider groups, no rider from the held-out test dataset contributed to model training or tuning. Within the two training datasets, the cross-validation folds were grouped by ride, ensuring that windows from the same recording session remained in the same fold. However, different rides from the same rider could occur in different internal folds. The internal resampling was therefore not fully rider-independent.

This LODO approach served two complementary goals. First, it reduced data leakage and thereby prevented overly optimistic performance estimates that can occur when the same or closely related observations contribute to both the training and test sets [30]. Second, it provided an exploratory assessment of model performance on held-out datasets. Performance metrics were computed for each configuration. Further details on the performance metrics are provided in Section 2.6.3.

Data from EPACs and conventional bicycles were pooled because the objective was to develop a common signal-based classifier for hard-braking and abrupt-swerving patterns across bicycle categories rather than separate bicycle-type-specific models. Bicycle type was therefore treated as a source of heterogeneity within the intended application. The controlled manoeuvres for both bicycle categories were conducted over the same speed range of 10–25 km/h, including the assisted operating range of the EPACs used in this study. However, bicycle type was not treated as a separate experimental factor, and the present results do not establish equivalent model performance for EPACs and conventional bicycles. The wider cycling population also includes other bicycle categories, such as cargo, mountain, and road bicycles, whose kinematic characteristics may differ. Establishing bicycle-type-specific performance would therefore require a substantially larger, deliberately stratified dataset and is outside the scope of this proof-of-concept study.

After the exploratory cross-dataset evaluation, the three main datasets (controlled track manoeuvres and the two urban datasets) were merged to fit a full-data model for possible future implementation. The 45 bicycle-only crash-like reference tests were not treated as a target class and were not used as positive training samples for hard braking or abrupt swerving. Instead, they were retained only as reference signals to compare low-speed impact-like kinematic patterns with those of the target manoeuvres.

#### 2.6.3. Offline Training and Evaluation of Stage 1 and Stage 2 Models

During offline model development, the Stage 1 and Stage 2 classifiers were trained and evaluated separately using the same labelled datasets. Stage 1 predictions were not used to select the training samples available to Stage 2. In the proposed future workflow, Stage 2 would be applied only to candidate windows identified by the Stage 1 trigger. The two stages were also evaluated sequentially on each held-out dataset.

A variety of statistical features, commonly used in time-series classification tasks [31], were extracted from shorter sliding windows within the six-second candidate windows to characterise the magnitude and variability of the sensor signals. These included the minimum, maximum, mean, and range (peak-to-peak) values of accelerations and angular velocities along the longitudinal, lateral and vertical axes.

Sliding windows of 1, 2 and 3 s were tested, each with 50% overlap. In the present study, these windows were used to describe the short, high-dynamic patterns associated with hard braking and abrupt swerving.

Four classification algorithms were considered:

(1) Decision Tree provides interpretability and relatively low computational load [32].
(2) Random Forest (RF), an ensemble of decision trees trained on randomised subsets of data and predictors, offers robustness and reduced overfitting [32].
(3) Support Vector Machine (SVM) handles non-linear relationships with the aid of kernel functions, making it robust in high-dimensional scenarios [32].
(4) Extreme Gradient Boosting (XGBoost) is a gradient boosting technique that refines predictions by iteratively correcting errors, featuring strong predictive accuracy and scalability [33].

The hyperparameters of each model were systematically tuned using grid search. These included, for example, maximum tree depth and learning rate for tree-based models and kernel parameters for the Support Vector Machine model. Tuning was performed using stratified five-fold cross-validation repeated five times, with grouping at the ride level. This repeated cross-validation procedure was used to reduce the variance of the performance estimates, as recommended by Kuhn and Johnson [34]. The sliding-window duration was also treated as a hyperparameter, with values of 1, 2 and 3 s tested. Stratified resampling was used to maintain the class distribution within each fold and to address the imbalance caused by the relative sparsity of the target manoeuvres.

The primary evaluation metric was the precision-recall area under the curve (PR-AUC). This metric summarises the trade-off between precision and sensitivity across all possible decision thresholds and was calculated as

(3) $$PR-AUC=\sum_{i=1}^{n-1}\left(s_{i+1}-s_{i}\right)\frac{p_{i}+p_{i+1}}{2},$$

where *p_i_* and *s_i_* denote precision and sensitivity, respectively, at the *i*-th decision threshold. Precision and sensitivity were calculated as

(4) $$Precision=\frac{TP}{TP+FP},$$

(5) $$Sensitivity=\frac{TP}{TP+FN},$$

where *TP*, *FP*, and *FN* denote true positives, false positives, and false negatives, respectively.

PR-AUC was selected because it is more informative than the area under receiver operating characteristic curve (ROC-AUC) for imbalanced datasets, where the negative class strongly dominates [35]. In such settings, ROC-based metrics can appear overly optimistic because the false-positive rate may remain small even when many false positives occur. By contrast, PR-AUC focuses on the performance for the positive class, which in this study corresponds to the target manoeuvres.

In addition, performance at the operating threshold was characterised by the F_1_-score, which balances precision and sensitivity (see Equation (6)). The operating threshold used for reporting sensitivity and precision was selected from the training data as the value that maximised the mean F1-score across the repeated cross-validation folds. This threshold was then applied unchanged to the held-out test data.

(6) $$F_{1}\;score=2\cdot \frac{Precision\cdot Sensitivity}{Precision+Sensitivity},$$

For the Stage 1 decision tree model, hyperparameters were chosen to maximise PR-AUC. For Stage 2, the RF, SVM and XGBoost models were compared, and the model with the highest PR-AUC was selected. To assess the complete two-stage workflow, an additional sequential evaluation was performed for each LODO configuration. Stage 1 was first applied to the complete held-out dataset, after which the selected Stage 2 model was applied only to windows flagged as candidates by Stage 1. Target windows not triggered by Stage 1 were counted as false negatives of the complete workflow. Both models and their hyperparameters were derived exclusively from the corresponding training datasets.

All analyses were performed in the R programming language using the tidymodels packages for consistent model fitting and evaluation [36].

## 3. Results and Discussion

### 3.1. Overview of Collected Kinematic Cycling Data

Window-level percentiles of the inertial sensor signals are reported in Table 4 by manoeuvre type to characterise the distribution of each sensor channel. The values were computed from 1.0 s sliding windows with 50% overlap, summarised within each dataset—track-controlled, urban A and urban B—and then aggregated as the median across datasets to provide a dataset-balanced summary. This window-based procedure reduced duration bias from long sequences and matched the feature-extraction procedure used in model training. To summarise the distributions in a concise and robust way, the 5th, 50th, and 95th percentiles were reported. The 50th percentile represents the median, while the 5th and 95th percentiles provide lower and upper distributional bounds that are less sensitive to isolated outliers. Gravity-compensated accelerations are denoted by ALX, ALY and ALZ for the lateral, longitudinal and vertical axes, respectively. Angular rates are denoted by GX, GY and GZ for rotations about the X, Y and Z axes, corresponding to roll, pitch and yaw. All axes were expressed in the bicycle’s frame of reference.

For non-target cycling behaviour, the distributions were centred around zero across all channels. The widest P95-P5 spreads were observed for the lateral acceleration axis, ALX, which is consistent with lane changes and curved riding, where lateral motion is expected. For hard braking, the longitudinal acceleration, ALY, was strongly negative, with a median near −2 m/s^2^ and a lower-tail value of −4.64 m/s^2^. The remaining channels stayed close to zero, showing that hard braking was mainly characterised by longitudinal deceleration rather than lateral or rotational motion. For abrupt swerving, the lateral acceleration, ALX, and the angular rates around the Y and Z axes, GY and GZ, showed wide P95-P5 spreads. The median longitudinal acceleration, ALY, was also moderately negative, indicating that some riders reduced speed while performing the lateral manoeuvre. Overall, abrupt swerving was mainly characterised by lateral acceleration and rotational motion, especially pitch and yaw, rather than by braking alone. The strongest signal magnitudes across all channels were observed in the bicycle-only crash-like reference tests. Despite the low approach speeds and the absence of a rider, these reference sequences showed signal ranges outside those observed for the target riding manoeuvres. They therefore provide useful reference signals for low-speed impact-like patterns, but they should not be interpreted as rider-involved crash dynamics.

The target manoeuvres lasted, on average, 2.7 s for hard braking and 2.5 s for abrupt swerving. These durations are compatible with the 1–3 s sliding windows evaluated during model training (see Section 2.6.3).

### 3.2. Model Performance on Held-Out Datasets

Table 5 summarises the model results by target manoeuvre type for each of the three training-test configurations defined in Table 3. In each configuration, models were trained on two datasets and evaluated on the third, held-out dataset. Hyperparameters were selected exclusively using the training datasets of each configuration.

Across configurations and model types, performance for hard braking was broadly comparable. PR-AUC values for the hard-braking models ranged from 0.842 to 0.995, whereas those for the abrupt-swerving models ranged more widely, from 0.616 to 0.965. The systematically lower and more variable performance for abrupt swerving may reflect greater variability within the class and increased overlap with non-critical lateral dynamics. Because abrupt swerves can range from sharp, short-radius deviations to more gradual lateral path adjustments to either side, this variability could complicate discrimination relative to the predominantly longitudinal signal patterns of hard braking. A similar asymmetry between longitudinal and lateral collision avoidance was reported by Li et al. [37], whose LIDAR-based trajectory model for e-scooters and bicycles underestimated lateral clearance in steering manoeuvres and could therefore lead to false negatives.

Within a given model family for hard braking classification, variability across the three LODO configurations was small: the PR-AUC spread (defined as the difference between the maximum and the minimum values) ranged from 0.07 to 0.12 for all families. This indicates reasonably consistent performance across the three exploratory held-out-dataset evaluations.

For abrupt-swerving, spreads were larger than for hard braking. The DT, RF and SVM models had PR-AUC spreads ranging from 0.09 and 0.16, whereas XGBoost showed greater variation, with a spread of 0.30. Notably, configuration 3 (trained on Urban A and Urban B; tested on track-controlled) yielded the highest PR-AUC values across most model families. This indicated that training on both urban datasets provided broader coverage of abrupt swerving variability, which resulted in improved performance. By contrast, configuration 1 performed worst for most models. Error analysis showed that barrier-navigation scenarios in the Urban B dataset were sometimes classified as abrupt-swerving target manoeuvres; this scenario appeared only once in Urban-A and not in the track-controlled dataset. This scenario was therefore under-represented in the training split and likely contributed to the lower performance. This imbalance was less pronounced in the full-data refit because the combined training pool included all recorded situations.

### 3.3. Kinematic Thresholds for Potentially Critical Cycling Manoeuvres

#### 3.3.1. Stability of Thresholds Across Datasets

In the offline evaluation, Stage 1 was represented by an interpretable decision tree model that yielded explicit kinematic thresholds for possible future on-device triggering. To assess the stability of these thresholds, the features and threshold values at the root and second-level splits were extracted for each dataset configuration.

Across the three dataset configurations, the decision tree for hard braking consistently used the minimum longitudinal acceleration (ALY) at the root split, with a median threshold of −2.94 m/s^2^ and a variation of only 0.35 m/s^2^. The second-level splits captured vertical vibration through the range of vertical acceleration (ALZ) in all configurations with a median threshold of 2.33 m/s^2^ and a variation of 0.58 m/s^2^. Hyperparameters were nearly identical across configurations: the complexity parameter was set to 1 × 10^−10^; the maximum depth was 2; the minimum terminal node size was 2; the window length was 2.0 s in one configuration and 3.0 s in the other two configurations, consistent with the average event durations. Physically, the root condition reflected the deceleration peak characteristic of hard braking, while the vertical amplitude constraint suppressed triggers caused by the road surface, e.g., cobblestones, potholes, and kerb strikes, that exhibit strong vertical vibrations without sustained longitudinal deceleration. Taken together, the consistent selection of the same features with tightly clustered thresholds across configurations indicated a stable rule set suitable for possible future on-device implementation.

For abrupt swerving, the root node in each decision tree used an amplitude-based feature, defined as the peak-to-peak amplitude within the sliding window. In two configurations, the root split was the lateral acceleration amplitude with the same threshold of 3.57 m/s^2^. In the third configuration, it was the yaw-rate amplitude with a threshold of 1.10 rad/s. This pattern is consistent with swerving manoeuvres that can manifest either through strong lateral acceleration or through dominant yaw rotation. The variation in the root feature across configurations likely reflects a combination of factors, such as riding style, speed, mounting variability, and surface-induced vibration, which cannot be disentangled with the present dataset. Larger naturalistic studies with a broader participant pool, additional cities, and more comprehensive alignment of sensors would be required to separate these influences. For possible future on-device implementation, a consensus rule was derived from a full-data refit (see Section 3.3.2). The second-level splits varied by configuration and comprised three variables: (i) the peak-to-peak longitudinal acceleration (<1.46 m/s^2^), (ii) the minimum pitch rate (≥−0.10 rad/s), and (iii) the range of yaw rate (≥1.00 rad/s). Physically, these second-level conditions acted as confounder filters: they suppressed segments with pronounced longitudinal dynamics, forward-pitch spikes induced by impacts, or alternative rotational patterns that can mimic swerving without intentional lateral avoidance. As with hard braking, the hyperparameters were nearly identical across configurations with a complexity parameter = 1 × 10^−10^, maximum tree depth = 2, minimum terminal node size = 2, window length = 2.0 s for two configurations and 3.0 s for one configuration, which indicated stable model structure across datasets.

#### 3.3.2. Consensus Thresholds Derived from the Full Dataset

For possible future on-device implementation, consensus thresholds were derived from a refit using the full dataset to capture all scenario variants. Because this refit includes the held-out domains, its diagnostic scores are likely to be overly optimistic and were therefore not used for performance estimation.

For hard braking, the consensus threshold rule is given by Equation (7):

Hard braking: min(ALY) < −2.78 m/s^2^ and range(ALZ) < 1.95 m/s^2^, (7)

with min(ALY) being the minimum longitudinal acceleration within the 2.0 s window and range(ALZ) being the peak-to-peak vertical acceleration within the window. This consensus decision tree closely matched the trees observed in the held-out model training (see Section 3.3.1): negative longitudinal accelerations were classified as hard braking only when the vertical acceleration remained small, which suppresses triggers caused by surface-induced artefacts, e.g., cobblestones, potholes, and kerb strikes.

For abrupt swerving, the consensus on-device rule is given in Equation (8):

Abrupt swerving: range(ALX) ≥ 3.71 m/s^2^ and range(ALY) < 1.47 m/s^2^, (8)

with range(ALX) being the peak-to-peak lateral acceleration within the 3.0 s window and range(ALY) being the peak-to-peak longitudinal acceleration within the window. This rule also followed the held-out trees closely: the first condition identifies windows with strong lateral motion, while the second condition acts as a filter for potential confounding signals that retains such windows only if they do not exhibit pronounced longitudinal accelerations.

#### 3.3.3. Comparison of Thresholds with Previous Studies

Relatively few bicycle-specific studies have defined explicit kinematic thresholds for detecting potentially critical cycling manoeuvres. For example, Dozza and Werneke [14] used kinematic triggers without reporting explicit numerical criteria, while Karakaya et al. [17] used heuristic rules to select the strongest dynamic movements in each ride rather than fixed thresholds. In motor-vehicle research, hard-braking events have often been defined using thresholds of approximately −5 m/s^2^ [29], which is stronger than the bicycle thresholds observed in the present study. This difference is reasonable because motor vehicles have different braking systems, higher operating speeds and more stable body dynamics than bicycles. The present results suggest that a threshold-based first-stage trigger can also be applied to bicycle sensor data when bicycle-specific kinematic patterns are used. However, the thresholds reported here were derived from controlled and semi-controlled data. They should therefore be interpreted as exploratory threshold values for possible future implementation and should be validated and, if needed, recalibrated in larger naturalistic cycling studies with more riders, bicycles, surfaces and traffic contexts.

### 3.4. Machine Learning Model for Classifying Potentially Critical Cycling Manoeuvres

#### 3.4.1. Selecting the Best Machine Learning Models

The Stage 2 model families were compared offline using the same held-out datasets. The proposed role of the Stage 2 models in a future workflow is to verify candidate windows identified by Stage 1 and thereby reduce false positives. Random Forest, Support Vector Machine and Extreme Gradient Boosting were compared. For this proof-of-concept study, the model family with the highest mean PR-AUC across the three leave-one-dataset-out configurations was selected for Stage 2. The F1-score was used as a complementary measure at the operating threshold. Table 6 reports the mean PR-AUC and F1-score for both manoeuvre types and all model families. The Stage 1 decision tree model represented the tuned threshold baseline against which the Stage 2 model candidates were compared using the same held-out datasets.

For hard braking, the Support Vector Machine achieved the highest mean precision–recall area under the curve, whereas the Random Forest achieved the highest mean F1-score. Because PR-AUC was the primary model-selection measure, the Support Vector Machine was retained for the subsequent Stage 2 evaluation. At the operating threshold, the mean precision across the three configurations was 0.943 for the Support Vector Machine and 0.825 for the decision tree. For abrupt swerving, the Support Vector Machine achieved the highest mean values for both measures.

For abrupt swerving, the mean PR-AUC and F1-scores were lower than for hard braking, but the ranking of the model families remained the same. The Support Vector Machine model performed best, followed by the Random Forest model, while Extreme Gradient Boosting showed lower performance. Both Random Forest and Extreme Gradient Boosting are tree-based ensemble methods. Random Forest aggregates many independently grown trees [32], whereas gradient boosting builds trees sequentially to correct previous errors [32]. Previous work has shown that the performance of gradient-boosting algorithms can depend substantially on hyperparameter settings [38], which may partly explain the weaker performance of Extreme Gradient Boosting in the present dataset. Because the Support Vector Machine model achieved the best overall performance for both target manoeuvre types, further optimisation of tree-based ensemble models was not pursued.

Compared with the Stage 1 decision tree, the SVM yielded a large PR-AUC gain for abrupt swerving (+0.228 absolute). At the operating threshold, precision increased from 0.402 for the DT to 0.880 for the SVM, reflecting a marked reduction in false positives. This suggests that the more flexible models were able to capture the more complex dynamics associated with swerving better than the simpler decision tree model. In the offline sequential evaluation, Stage 1 acted as a high-sensitivity screening rule for candidate high-dynamic patterns, and Stage 2 filtered these candidates before downstream processing. The Stage 2 Support Vector Machine achieved higher precision than the Stage 1 decision tree when compared using the same held-out datasets. This supports its proposed role as a verification model in the two-stage workflow. The sequential performance of the complete workflow is reported in Section 3.4.3.

#### 3.4.2. Hyperparameter Tuning

After selecting SVM as the best-performing Stage 2 model family (see Section 3.4.1), hyperparameters were tuned separately for hard braking and abrupt swerving using cross-validation within the LODO training configurations. In addition to the length of the sliding window, three SVM model parameters were optimised. Table 7 lists the tuned parameters, the tested ranges, and the selected consensus values for the SVM models for hard braking and abrupt swerving.

**Figure 6:**
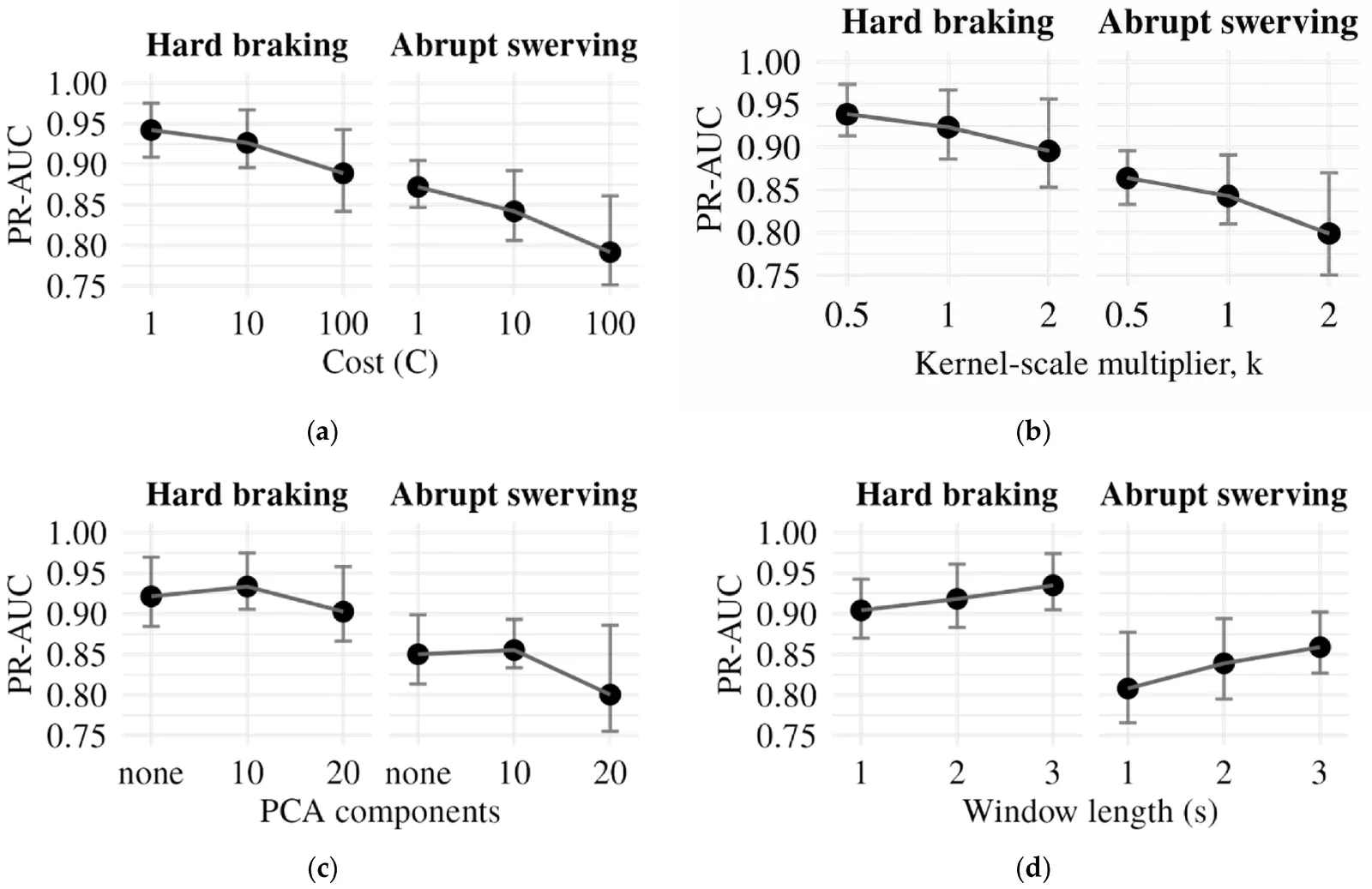
Effects of SVM hyperparameter tuning: (**a**) cost parameter, C; (**b**) kernel-scale multiplier, k; (**c**) number of PCA components; (**d**) length of the sliding window (s). Points show mean PR-AUC across cross-validation folds and LODO configurations; error bars show the interquartile range (25th and 75th percentiles).

Figure 6 shows the effects of the four Support Vector Machine hyperparameters, averaged over the cross-validation folds and leave-one-dataset-out training configurations. Across both target manoeuvre types, the patterns were consistent: the mean precision-recall area under the curve was highest with moderate regularisation, C = 1, the smaller kernel scale, k = 0.5, and longer sliding windows of 3.0 s. The effect of principal component analysis was limited because using 10 components produced only small gains compared with using the normalised predictors without dimensionality reduction.

For the full-data refit, a single consensus configuration was selected by maximising the minimum cross-validated precision-recall area under the curve across the three configurations. This criterion was used to select a configuration that performed consistently across datasets. The selected settings were the same for both target manoeuvre types for the cost parameter, C = 1, the kernel scale, k = 0.5, and the sliding-window length, 3 s. The only difference was the use of principal component analysis with 10 components for hard braking, while no dimensionality reduction was used for abrupt swerving. This choice is consistent with the limited effect of principal component analysis observed in Figure 6 and may reflect differences in feature collinearity between the two manoeuvre types. For hard braking, several predictors, such as longitudinal deceleration and vertical signal variability, were partly redundant, and principal component analysis may therefore have reduced redundant information [40].

#### 3.4.3. End-to-End Performance of the Two-Stage Workflow

The end-to-end evaluation assessed the complete sequential workflow by applying Stage 1 to the entire held-out dataset and then applying Stage 2 only to windows flagged as candidates. Table 8 summarises the number and class composition of the candidate windows passed to Stage 2 for each LODO configuration. Table 9 then reports the resulting performance changes relative to the Stage 1-only baseline.

To quantify the effect of Stage 2 on false-positive detections, the relative false-positive reduction was calculated as

(9) $${FP}_{reduction}=\frac{{FP}_{stage1}-{FP}_{two\_stage}}{{FP}_{stage1}}\times 100\%,$$

where *FP_Stage_* 1 denotes the number of false-positive detections produced by Stage 1 and *FP_two_stage_* denotes the number of false-positive detections remaining after Stage 2 verification. A value of 0% indicates that Stage 2 did not remove any false positives, whereas a value of 100% indicates that all Stage 1 false positives were removed.

Stage 2 reduced false-positive detections in every configuration. The relative reduction ranged from 20.0% to 83.3% for hard braking and from 87.2% to 98.2% for abrupt swerving. Sensitivity decreased by up to 0.08 for hard braking and 0.12 for abrupt swerving. The final two-stage F1-scores ranged from 0.914 to 0.967 for hard braking and from 0.783 to 0.937 for abrupt swerving.

### 3.5. Comparison to Existing Studies and Implications for Naturalistic Applications

This study used conventional machine learning models, namely Random Forests, Support Vector Machines and Extreme Gradient Boosting, rather than neural networks. This choice was made because the dataset of labelled target manoeuvres was relatively small, which increased the risk of overfitting when using high-capacity models. In contrast, Karakaya et al. [18] used neural networks on a large naturalistic cycling dataset collected with smartphones. More recently, Saleh et al. [19] proposed a deep temporal model evaluated using the same data source. Both studies addressed binary detection of self-reported near-miss events from smartphone IMU and GPS data. Their work demonstrates the potential of deep learning for naturalistic near-miss detection and, in the case of Saleh et al. [19], the feasibility of reducing model complexity. Whereas Saleh et al. investigated a computationally efficient model with potential for on-device near-miss classification, the proposed workflow uses an explicit lightweight trigger to select short candidate windows before more detailed classification. This design provides interpretable triggering criteria and is intended to reduce the need for continuous storage and transmission of high-frequency sensor data.

Karakaya et al. [18] also reported reduced performance when models were transferred across regional subsets, which illustrates the challenge of generalising models trained on heterogeneous crowdsourced data. The present approach achieved high precision within its own evaluation, despite being developed using a substantially smaller dataset. However, direct numerical comparison with the approaches of Karakaya et al. [18] and Saleh et al. [19] is not appropriate because the target definitions, annotation procedures, class compositions and validation strategies differ.

Beyond differences in model architecture, these studies also underline the importance of label quality in supervised classification. Both Karakaya et al. [18] and Saleh et al. [19] relied on near-miss events reported by cyclists after their rides, which can introduce uncertainty in the event definition and timing, especially for manoeuvres lasting only a few seconds. In the present study, this source of uncertainty was reduced by using clearly defined target manoeuvres and synchronised annotations. Related studies have also explored unsupervised or weakly supervised methods to detect anomalies in cycling data as proxies for safety-relevant events. For example, Yaqoob et al. [41] used deep transfer learning to detect and localise anomalies in cycling behaviour from Global Navigation Satellite System data. Because their data, labels and validation procedure differed from those used here, direct numerical comparison is not appropriate.

Overall, the findings highlight the trade-off between sensitivity and precision in the proposed two-stage workflow. The method will not capture every safety-relevant event, especially situations without a clear evasive kinematic response. Nevertheless, it can support proactive safety screening by providing repeated observations of selected high-dynamic manoeuvres, such as hard braking and abrupt swerving. In large naturalistic cycling studies, aggregated detections may help identify locations where these manoeuvres occur more frequently and where further assessment may be useful. Large-scale smartphone-based studies of hard-braking events in passenger cars have shown that aggregated kinematic events can be related to crash patterns at the network level [29]. Recent research using crowdsourced bicycle accelerometer and GPS data has similarly shown that near-crash observations can reveal infrastructure-related risk patterns, although the factors associated with near-crashes and reported crashes may differ [16]. This underlines the value of crowdsourced near-crash data as a complementary source of safety evidence rather than as a substitute for crash records. At the same time, the differences between near-crash and crash-related risk factors reinforce the need to validate sensor-based detections against independent evidence, such as video-based conflicts, site inspections or police-recorded crashes.

Nevertheless, the relatively small participant pool and the controlled conditions in this study may limit the generalisability of the results. Future work should therefore validate the method under fully naturalistic conditions and with a larger, more diverse group of cyclists to confirm its robustness and applicability on a larger scale.

The proposed workflow should be viewed as complementary to interaction-based surrogate safety measures such as TTC and PET, rather than as a direct substitute. TTC and PET characterise the temporal proximity between road users and therefore provide information about the interaction associated with a potentially critical situation. In contrast, the present approach detects only the kinematic response of the bicycle and cannot identify the cause of that response from these data alone. Its principal advantage is the potential to screen larger road networks without requiring camera installations at every location. Targeted video analysis could subsequently provide the contextual information needed to assess road-user interactions using measures such as TTC or PET. Future studies could combine both approaches by validating smartphone-detected manoeuvres against video-derived interaction measures at selected locations.

To deploy the proposed workflow in a naturalistic study, further validation beyond the standardised setup used here would be required. In particular, both the Stage 1 thresholds and the Stage 2 classifiers should be evaluated across a broader range of riders, bicycle types, road surfaces, weather conditions, smartphone models and mounting orientations. Larger studies may also reveal additional sources of false-positive detections that were not represented in the present dataset, such as lifting or handling the bicycle, which may produce high accelerations or angular velocities. Less controlled smartphone mounting would further require robust three-axis orientation alignment. Moreover, long-term field studies would require a practical application that participants are willing to use regularly. Future work should therefore assess on-device computational and battery demands, the volume of candidate windows transmitted to the server, application usability and participant adherence over extended recording periods.

### 3.6. Limitations and Scope of Inference

The main limitation of this study is that most target manoeuvres were controlled or rider-initiated proxy manoeuvres rather than confirmed real-world traffic conflicts. Only three hard-braking manoeuvres in the urban datasets were genuine traffic-related events. Therefore, the reported model performance reflects the detection of signal-defined hard-braking and abrupt-swerving patterns, not the direct detection of near-crashes as complete traffic events.

The method also relies only on ego-bike kinematics. It can detect manoeuvres with a clear braking or swerving response, but it cannot capture safety-relevant situations without a clear evasive movement, such as close passes without rider reaction. The sample size was also limited to 10 riders, including project-team riders, and the smartphones were mounted in a controlled handlebar position. Performance may change with a larger rider population, other bicycle types, different surfaces, or less controlled phone placement.

Although each outer held-out dataset contained entirely unseen riders, internal hyperparameter tuning was grouped by ride rather than by rider. Different rides from the same rider could therefore occur in different internal folds. This may have favoured rider-specific parameter selection. The results should consequently be interpreted as exploratory cross-dataset estimates rather than as independent external validation.

The same three held-out datasets were used for the exploratory comparison of model families. Therefore, selection of the Support Vector Machine and its reported performance are not statistically independent.

The bicycle-only crash-like reference tests were performed with an unoccupied bicycle at low speed and were not intended to represent rider-involved crash dynamics. They were used only as reference signals for impact-like kinematic patterns. Future work should validate the workflow under fully naturalistic conditions and compare detected manoeuvres with independent safety evidence, such as video-based conflicts, site inspections, complaints, or crash records.

## 4. Conclusions

This paper presented a proof-of-concept two-stage workflow for offline detection of selected potentially critical cycling manoeuvres using smartphone sensor data. The data were collected in controlled test-track scenarios and urban rides and used for exploratory cross-dataset evaluation. In the offline analysis, a threshold-based method identified candidate high-dynamic manoeuvres, and machine learning models classified these candidate windows to reduce false positives. The following workflow is proposed for possible future integration into a smartphone application with a cloud-based server backend:

- The collected smartphone sensor data demonstrated the feasibility of detecting selected high-dynamic cycling manoeuvres, especially hard braking and abrupt swerving. These manoeuvres can serve as surrogate safety indicators, provided that their safety meaning is validated with contextual information such as video observations, site inspections or crash records.
- The offline sequential evaluation of the two-stage design reduced false positives in every held-out dataset. Final precision ranged from 0.923 to 0.970 for hard braking and from 0.754 to 0.981 for abrupt swerving, with sensitivity decreases of up to 0.08 and 0.12, respectively.
- In future naturalistic cycling studies, this approach could support network-level safety screening without relying on specially equipped bicycles. Locations with repeated hard-braking or abrupt-swerving manoeuvres should be interpreted as candidate locations for further assessment, for example through field inspection, video-based conflict analysis or comparison with reported crash data.
- In a future implementation, the two-stage design could support data minimisation by transmitting only short candidate manoeuvre windows rather than continuously streaming video data or full high-frequency trajectories. This could reduce storage needs and privacy concerns and may support user acceptance in larger cycling studies.
- Automated sensor-based manoeuvre detection can reduce subjective timing errors associated with user self-reports and support consistent large-scale collection of selected surrogate safety indicators. Further validation is needed before these indicators are used as direct measures of infrastructure-related crash risk.

Despite these strengths, the dataset was collected from a limited number of participants under controlled and semi-controlled conditions. Most target manoeuvres were controlled or rider-initiated proxy manoeuvres, and only a small number were genuine traffic-related events. Future research should therefore validate the workflow under fully naturalistic conditions with a larger and more diverse group of cyclists. Detected manoeuvres should also be compared with independent safety evidence, such as video-based traffic conflicts, site inspections, complaints, or crash records, before they are used as direct indicators of infrastructure-related safety risk.

## 5. Practical Applications

After prospective field and real-time validation, the proposed workflow could be developed into a low-burden screening tool for cycling safety programmes. By recording only short sensor windows around high-dynamic manoeuvres, the method could help road authorities identify corridors, junctions or cycling facilities where hard braking or abrupt swerving occurs repeatedly. These locations should not be interpreted as confirmed crash hotspots based on sensor data alone. Instead, they should be treated as candidate sites for further investigation, such as field inspection, video-based conflict analysis, review of infrastructure geometry, or comparison with reported crashes and complaints. In this way, the method can complement crash-based safety management, especially where cycling crashes and falls are underreported.

## Figures and Tables

**Table 1:** Overview of datasets used for model development and exploratory cross-dataset evaluation.

Dataset	Environment	City	Rider Count	Hard Brakings	Abrupt Swervings	Bicycle-Only Crash-like Reference Tests
Track-controlled	Test track	-	4	58	58	45
Urban A	Naturalistic urban (including rider-initiated manoeuvres)	Aachen	3	53	48	0
Urban B	Naturalistic urban (including rider-initiated manoeuvres)	Bonn	3	37	26	0
Combined	-	-	10	148	132	45

Note: Only three hard-braking manoeuvres in the urban datasets were genuine traffic-related events. All other target manoeuvres were controlled or rider-initiated proxy manoeuvres. Bicycle-only crash-like reference tests were not used as target classes.

**Table 2:** Classification of cycling manoeuvres with observable kinematic patterns.

Class	Event Type	Description
Target manoeuvre	Hard braking	Abrupt deceleration pattern that can occur during evasive braking.
	Abrupt swerving	Sudden lateral deviation pattern that can occur during evasive steering.
Non-target manoeuvre	Braking	Braking with foresight, e.g., before a traffic light.
	Turning	Turning left onto another street or sidewalk.
	Starting	All movements required to move the bike straight ahead from rest.
	Going straight	Riding along the road or cycle path, including curved sections.
	Lane changing	Changing lanes or moving aside to avoid foreseeable obstacles.
	Waiting	Pausing without moving forward.

**Table 3:** Training and test set configurations for exploratory cross-dataset evaluation.

Config. ID	Training Set	Test Set	Training Target Manoeuvres Hard Braking/Abrupt Swerving	Test Target Manoeuvres Hard Braking/Abrupt Swerving
1	Track-controlled + Urban A	Urban B	111/106	37/26
2	Track-controlled + Urban B	Urban A	95/84	53/48
3	Urban A + Urban B	Track-controlled	90/74	58/58

Note: The values refer only to the two target manoeuvre types, hard braking and abrupt swerving. Bicycle-only crash-like reference tests were not included as target manoeuvres.

**Table 4:** Summary of sensor signal distributions by manoeuvre type. P5, P50 and P95 denote the 5th, 50th (median) and 95th percentiles, respectively.

	Non-Critical			Hard Braking			Abrupt Swerving			Crash Test Reference		
Sensor	P5	P50	P95	P5	P50	P95	P5	P50	P95	P5	P50	P95
ALX	−0.81	0.00	0.81	−0.59	0.05	0.48	−3.03	−0.29	2.16	−4.69	−0.15	4.55
ALY	−0.67	−0.06	0.56	−4.64	−2.00	0.20	−0.80	−0.32	0.15	−4.89	−0.31	2.87
ALZ	−0.30	0.01	0.32	−0.17	0.04	0.27	−0.39	0.31	1.22	−4.42	−0.24	1.31
GX	−0.04	0.00	0.07	−0.05	0.00	0.04	−0.02	0.11	0.27	−0.48	−0.04	0.16
GY	−0.20	0.00	0.19	−0.08	0.01	0.14	−0.58	0.02	0.73	−0.82	0.07	1.92
GZ	−0.28	0.00	0.25	−0.22	−0.01	0.19	−0.77	0.03	0.89	−1.01	−0.02	0.46

Units: acceleration (ALX, ALY, ALZ) in m/s^2^; angular velocity (GX, GY, GZ) in rad/s.

**Table 5:** Model performance on the held-out test sets by target manoeuvre type across the three leave-one-dataset-out configurations. Configuration identifiers correspond to the configurations in Table 3. Sensitivity and precision are reported at the operating threshold selected using the training data.

Model	Config. ID	PR-AUC		Sensitivity		Precision	
		Hard Braking	Abrupt Swerving	Hard Braking	Abrupt Swerving	Hard Braking	Abrupt Swerving
DT	1	0.883	0.647	0.919	0.808	0.850	0.429
	2	0.842	0.616	0.981	0.938	0.703	0.292
	3	0.960	0.707	1.000	0.914	0.921	0.486
RF	1	0.896	0.776	0.917	0.769	0.917	0.645
	2	0.946	0.933	0.925	0.854	0.925	0.911
	3	0.995	0.911	1.000	0.845	0.935	0.961
SVM	1	0.923	0.811	0.838	0.769	0.969	0.870
	2	0.951	0.877	0.906	0.938	0.873	0.789
	3	0.989	0.965	0.983	0.931	0.987	0.982
XGB	1	0.876	0.621	0.892	0.692	0.786	0.581
	2	0.943	0.873	0.943	0.854	0.909	0.759
	3	0.896	0.925	1.000	0.879	0.935	0.944

**Table 6:** Mean model performance by target manoeuvre type and model family.

Target Manoeuvre Type	Model	Mean PR-AUC	Mean F1-Score
Hard braking	Decision Tree	0.895	0.887
	Random Forest	0.946	0.936
	Support Vector Machine	0.954	0.924
	Extreme Gradient Boosting	0.905	0.909
Abrupt swerving	Decision Tree	0.657	0.547
	Random Forest	0.873	0.827
	Support Vector Machine	0.884	0.876
	Extreme Gradient Boosting	0.806	0.782

Values are unweighted arithmetic means across the three held-out datasets. The F1-score was calculated separately for each configuration from the corresponding sensitivity and precision and was then averaged.

**Table 7:** Tuned SVM hyperparameters, search ranges and selected consensus values.

Parameter	Description	Tested Values	Selected Value (Hard Braking)	Selected Value (Abrupt Swerving)
Cost	Regularisation parameter	1, 10, 100	1	1
Kernel scale	Width parameter for the radial basis function kernel, based on the heuristic method of [39]	0.5, 1, 2	0.5	0.5
PCA components	Optional dimensionality reduction applied to normalised predictors prior to training	none, 10, 20	10	none
Sliding window	Duration of the sliding window in seconds to compute features	1, 2, 3	3	3

**Table 8:** Class composition of Stage 1 candidate windows in each LODO configuration.

Manoeuvre Type	Config.	Target Manouevres	Candidates	True Positives	False Positives	False Negatives
Hard braking	1	37	40	34	6	3
	2	53	74	52	22	1
	3	58	63	58	5	0
Abrupt swerving	1	26	49	21	28	5
	2	48	154	45	109	3
	3	58	109	53	56	5

**Table 9:** Stage 1 and two-stage performance across the leave-one-dataset-out configurations.

Manoeuvre	Config.	Stage 1 Counts	Two-Stage Counts	Stage 1 Metrics	Two-Stage Metrics	FP_reduction_
Hard braking	1	34/6/3	32/1/5	0.919/0.850/0.883	0.865/0.970/0.914	83.3%
	2	52/22/1	48/4/5	0.981/0.703/0.819	0.906/0.923/0.914	81.8%
	3	58/5/0	58/4/0	1.000/0.921/0.959	1.000/0.935/0.967	20.0%
Abrupt swerving	1	21/28/5	18/2/8	0.808/0.429/0.560	0.692/0.900/0.783	92.9%
	2	45/109/3	43/14/5	0.938/0.292/0.446	0.896/0.754/0.819	87.2%
	3	53/56/5	52/1/6	0.914/0.486/0.635	0.897/0.981/0.937	98.2%

Counts are reported as true-positive/false-positive/false-negative detections, respectively. Metrics are reported as sensitivity/precision/F1-score. FP_reduction_ is calculated relative to Stage 1.

## Data Availability

The data presented in this study are not publicly available due to privacy restrictions associated with potentially identifiable participant data.
